# Social and Behavioral Health Factors Associated with Violent and Mature Gaming in Early Adolescence

**DOI:** 10.3390/ijerph17144996

**Published:** 2020-07-11

**Authors:** Linda Charmaraman, Amanda M. Richer, Megan A. Moreno

**Affiliations:** 1 Wellesley Centers for Women, Wellesley College, Wellesley, MA 02481, USA; aricher@wellesley.edu; 2 Department of Pediatrics, University of Wisconsin-Madison, Madison, WI 53705, USA; Moreno@wisc.edu

**Keywords:** violent gaming, problematic gaming, adolescence, social interactions, behavioral health

## Abstract

We examined how risk level of video games, measured by maturity and violence level, was associated with behavioral health, social impacts, and online social interactions. School-based surveys in two different cohorts assessed self-reported gaming behaviors, health, and social media use. For Study 1, our 700 participants were 52% female and 48% White (mean age 12.7). Middle school students who played the high-risk games reported higher depressive symptoms and problematic internet behaviors, less sleep, more time spent playing games, and higher frequency of checking social media than non-gaming students. Those who played high-risk games were less likely to play alone and to play with strangers than those who played minimal-risk games. For Study 2, our 772 participants were 50% female and 57% White (mean age 12.6). Similar to Study 1, we found that those who played the high-risk games spent significantly more time playing games, were more interactive with other players, and had poorer sleep outcomes than non-high-risk gamers. Additionally, playing high-risk games had significantly different social impacts of gaming compared to less-risky gaming, including spending more money on games, spending less time on homework and with family or skipping meals due to gaming. Mature and violent content of video games and amount of online social interaction associated with gaming play a strong role in behavioral health and social impacts within families. These results can inform guidelines to intervene when problematic behaviors emerge.

## 1. Introduction

According to a recent Pew Research Center study [1], 90% of US teens play games of some kind, such as on a game console, computer, or cell phone. In fact, the majority of adolescents in the U.S. and other developing countries play video games [2], which, at the low and moderate gaming levels, can provide social benefits for some adolescents [3], such as coping strategies and fulfilling social needs [4]. At the opposite end, higher gaming levels can result in excessive online gaming for others. With the inclusion of the Internet Gaming Disorder (IGD) as an “emerging” disorder in the Diagnostic and Statistical Manual for Mental Disorders 5 [5] and Gaming Disorder in the WHO’s ICD-11 [6], questions still remain about how to determine positive, non-pathological heavy gaming from problematic gaming that interferes with one’s daily life [7]. Internet gaming disorder (IGD) is associated with a multitude of socioemotional issues which can interfere with academic achievement and developing healthy social interactions [8], including depression, anxiety, self-esteem problems, high antisocial behavior, aggressive thoughts, lack of empathy, anger control problems, hyperactivity/inattention, and clinically significant reduction in sleep quality [4,9,10,11,12,13]. Adolescents who have attention problems and are socially vulnerable (i.e., lack social skills or struggle with social relationships) are at a higher risk of developing IGD symptoms [4]. Several studies have shown gendered patterns in vulnerability of developing problematic gaming behaviors [2,4,9]. For instance, boys who are lonely and socially anxious are significantly more likely to adopt maladaptive gaming behaviors in the absence of positive online social interactions [7].

As opposed to examining social and behavioral associations with pathological gaming that would classify an early adolescent as having IGD (APA) or Gaming Disorder (WHO), we are focused on problematic gaming behaviors that may or may not surpass these suggested clinical thresholds, turning our attention to a non-clinical, school-based sample ranging from no gaming at all to high levels. Besides identifying the risk factors that are associated with problematic and engaged but non-pathological gaming, it is also critical to consider the content of games consumed for vulnerable populations. According to the Pan European Game Information organization, forty percent of video games that were present in the 2016 market were deemed inappropriate for tweens and young teens due to exposure to violence, strong language, blood, and gore. The vast majority of games (89–96%) that are rated for teens or more mature adult players have been found to be explicitly violent [14,15]. However, there are limitations to relying on commercially-available ratings of violent content [16], especially when they usually focus on recommendations for teens in general, not tween and early teen years, when parents are less familiar with how to supervise transitions into more mature gaming content. Early adolescents have been shown to be particularly vulnerable to the effects of violent content during this stage of social, emotional, cognitive, and neurological development [17]. By focusing on only M-rated games, there is a potential for substantially underreporting children’s and young teens’ exposure to age-inappropriate gaming content [16]. Prior studies have utilized various techniques to classify content of games, such as assessing exposure by checking whether participants ever played one of four researcher-identified popular games [18], categorizing violent gaming content according to one externally-derived measure such as the ESRB rating [16], or allowing teens to evaluate their own exposure to violent gaming content for their favorite games [19]. The current study developed a protocol to move beyond the entertainment industry-driven ratings and include other criteria beyond violent content, such as sexual themes, substance use, and language used in chat rooms.

A major concern around problematic gaming behaviors is whether the excessive time spent displaces real-life social interactions and whether that is necessarily detrimental or simply “engaged” and for whom [7]. While teen gaming can be a solitary activity, teens often play with friends they know in person (89%) or met online (54%), or strangers (52%) [20]. A majority of teen gamers (78%) report that playing interactive online games makes them feel more connected to friends they already know [20]. Online gaming has been shown to provide a key way to maintain existing friendships [21], make new social connections [22], and expand sources of social support for shy adolescents [23]. Through the *social compensation* hypothesis [21], adolescents with difficulty in face-to-face social competence can use the safety net of online interactions to compensate for their real-life social inadequacies. For instance, adolescents with IGD have been found to over-rely on gaming to bolster their self-esteem and gain social acceptance from peers [24]. In contrast, for adolescents who are already successfully navigating their well-developed social skills, online gaming can extend that social capital [25] in what is known as the *augmentation hypothesis* [26]. Not only do the “rich get richer” in this scenario, but the poor get poorer, in that those who experience anxiety, depression, or low academic achievement can immerse themselves in gaming as a way to escape from their troubles, which can lead to compulsive online gaming over time [27].

Prior studies on the social outcomes of problematic gaming in adolescents have primarily focused on proximal impacts on school achievement, such as being highly associated with a worse grade point average or gaming-related school truancy [2,28]. Prior research has focused on *quantity* of gaming that has detrimental effects on academic achievement, whereas research on the effects of the *content* of gaming is often associated with negative affect, such as the relationship between violent gaming and aggression [19]. Relatively speaking, fewer studies have addressed how problematic gaming impacts family relations, particularly given the critical socialization role of parents in childhood and adolescence [29]. In the few studies that exist, problematic gaming was related to lower-quality family relationships and greater family conflict [30], and poor-quality parent–child relationships can exacerbate the severity of problematic gaming. A recent review revealed that a strong paternal bond can be protective of problematic gaming [31]. Poorer family functioning has also been associated in households with problematic gaming, including higher parental anxiety due to the antisocial behavior and anger management issues of their adolescents with IGD [9,32]. Another issue that arises in households with problematic gaming behaviors is the larger amounts of spending on free-to-play games compared to households with non-problematic gamers [33], which may lead to family conflict. One study found weak relationships between problematic gaming and slightly lower parental closeness and family conflict, but maintained that gaming frequency and playing with online strangers was a much stronger predictor of gaming problems than parent–child dynamics [34]. More research is needed to understand the role of parent–child relationships and the content (not just quantity) of adolescent gaming.

Prior studies have emphasized quantity of gaming, that is, frequency of gameplay or number of games played, but few studies have examined social and behavioral health outcomes associated with playing games with varying levels of violent or age-inappropriate *content*. Previous research is more limited in how playing violent and mature games is related to online social interactions and how gaming affects daily life in the home and school contexts. The purpose of the following two studies was to understand the association between playing violent or age-inappropriate online games and early adolescent behavioral health outcomes in two different community cohorts—the first sample was characterized by having a relatively low to medium gaming risk, whereas the second sample had a fairly higher risk gaming profile. In Study 1, we hypothesize that gaming risk level will be associated with problematic internet behaviors and increased social interactions while gaming, particularly with unknown players. In Study 2, we hypothesize that the higher the risky gaming content, the more likely participants would experience evidence of social withdrawal. By using two different methods of determining how often early adolescents are exposed to risky gaming content, we hypothesize that behavioral outcomes such as sleep or depressive symptoms will be associated with risky gaming content in both middle school-based samples in nuanced ways depending on how it was measured.

## Study 1

## 2. Materials and Methods

### 2.1. Data Collection

Study 1 was part of a larger cross-sectional study conducted in the Northeast US examining early adolescent social media use in the period 2017–2018. The study was named the Learning, Social Media, and Healthy Behaviors Project. We invited schools within one hour’s drive from our campus that represented a range of social contexts in terms of school size, urbanicity, race/ethnicity, and socioeconomic status. Following approval by the Wellesley College IRB Committee and school districts, principals from a small urban middle school, a large suburban middle school, and an urban afterschool program provided access to a convenience sampling of their 6th–8th grade students for a 40 min online survey during a designated period. Parents were informed of the study and the availability of a sample student survey to review through emails sent by school liaisons. Through a waiver of consent documentation process approved by our IRB, parents were given the opportunity to opt their child out of participating in the study. Schools provided laptops to take the voluntary Qualtrics survey and students provided online assent before taking the survey. On the survey date, each participating classroom had a researcher proctor the online survey by reading the assent information explaining the goals, procedures, risks, benefits, and voluntary nature of the study. All students who were present during the survey administration were provided an embossed pen as an incentive to participate. Survey takers were also entered into a raffle for gift card incentives. Schools were provided with a report of aggregated results and an honorarium in appreciation for their participation in the study.

### 2.2. Measures

Sociodemographic variables used as covariates included gender, age, and free/reduced-price lunch. Age was calculated by subtracting students’ self-report date of birth from the date of survey administration. Students were able to identify their gender as male (1) female (0) or “Other.” Students who identified as “Other” were recoded to missing given the extremely small prevalence. Students indicated whether they received free/reduced-price lunch through yes (1) or no/don’t know (0) responses.

*Gaming risk level* was assessed by asking survey participants to indicate all of the online games that they had ever played from a checklist of multiple games. Prior to data collection, the research team conducted a scan of the most popular games as of late summer 2017. Since most available statistics referred to the games that adults aged 18 and over played, we finalized the list of 16 games based on our pilot study of three early adolescents who test drove the survey and provided feedback on the relevance and acceptability of the answer choices. Respondents could also fill in additional games that were not listed. All possible online games that respondents played were evaluated by undergraduate research assistants to assign a risk level value to each game. Before conducting any statistical analyses, we conducted this separate recoding process of independently assigning a risk level to games that were stripped of any identifying data pertaining to who played those games in our dataset. The Education Software Rating Board (ESRB) rating and Common Sense Media’s ratings (from polling parents) were taken into account in determining level of risk. Suggested age limits from Common Sense parents and ESRB were only one measure of our researcher-defined gaming risk levels, since they each had their own systems for determining what was age appropriate. The ESRB includes 17 content descriptors that are rated E for appropriate for everyone (the most frequently assigned rating by ESRB), E10+ (for ages 10 and up), Teen (for ages 13+), and Mature (for ages 17+). The descriptors that we focused on pertained to blood/gore, violence, language, nudity, sexuality, and substances. We also took into account how users tend to interact with one another while gaming, such as being exposed to unfiltered and uncensored user-to-user communications via social media. Risk levels were coded as 0 if the participant indicated being a non-gamer. A risk level of 1 was considered “minimal risk,” including non-violent, age-appropriate games (e.g., Minecraft, Temple Run) that were often rated E or E10+. A risk level of 2 was considered “moderate risk,” including borderline age-appropriate games with some violence (e.g., Fortnite and Terraria) and may contain games rated as Teen for ages 13+. The highest risk level of 3 includes non-age-appropriate games with gory violence (e.g., Grand Theft Auto and Fallout) and medium to high levels of other mature descriptors such as profanity, substances, or sexuality. All games rated as Mature fell into this highest risk category. All games were independently coded by two researchers during the initial open coding phase, and then, during the secondary phase, all disagreements (2%) were reviewed until consensus. See Appendix A for a coding scheme for game risk level.

#### 2.2.1. Behavioral Health

*Depression symptoms*. We used the 4-item Center for Epidemiological Studies Depression Scale for Children (ages 10–13; CES-DC): “Please check how much you felt this way in the past week:” I was happy, I felt that friends didn’t want to be with me, I felt sad, It was hard to get started doing things, etc. This scale has been shown to have a Cronbach’s alpha of 0.583 [35]. We added two additional items from the CESDR-10 which have been validated on older adolescent samples (aged 13–18): could not focus on the important things and felt irritable or cranky, with a Cronbach’s alpha of 0.90–0.91 (4-point Likert scale, CESD, [36]). The alpha for the current sample was 0.79.

*Sleep duration*. Participants were asked on average how many hours of sleep they get on a typical school night. Responses ranged from 4 h or less to 10 or more hours. Average hours of sleep for participants was 8 h.

*Recreational computer use*. Participants reported how many hours they use the computer (or other screen device) for doing something other than school work. Responses were reported on a 7-point scale ranging from “none” to “5 or more hours per day”. On average, participants reported using the computer for 1–2 h per day.

*Problematic internet behaviors*. Using the 3-item Problematic and Risky internet Use Screening Scale (PRIUSS) [37], participants reported how often they lose motivation to do other things because of the internet, feel nervous or anxious when not online, or become moody or depressed when not online using on a 5-point scale of “never” to “very often” (alpha = 0.70). The average problematic internet behaviors for the sample was 1.78 (SD = 0.70), which is between “never” and “rarely”.

*Online gaming frequency*. Students reported how often they played online games on a typical school day. Responses were reported on a 5-point scale ranging from “I don’t play online games” (lowest value) to “3 or more hours” (highest value). The average online games play time for the full sample was 2.39 (SD = 1.27), which was equivalent to an average of up to 60 min play on a typical school day.

#### 2.2.2. Online Social Interactions

*Online gaming interactions*. Participants who indicated that they played online games were asked whether they (a) usually play alone or against no one (54%) or (b) play with strangers (i.e., “I have no idea who I play with online”; 23%).

*Social media frequency*. Frequency of checking social media on a typical school week was measured on a 7-point scale ranging from “never” (lowest value) to “more than every hour” (highest value). The average social media checking frequency for the full sample is 2.48 (SD = 1.48), suggesting that the average participant checks social media between every few days to once a day.

Refer to Appendix B for a list of self-created measures.

### 2.3. Data Analysis

All analyses were conducted in SPSS version 25 (IBM SPSS Statsistics for Windows, Armonk, NY, USA). For the power analyses to estimate the sample size, we used *p* = 0.05 and power = 0.80 as target values. For a sample size of at least 700, for linear regression models estimating associations, we estimated that individual regression parameters as low as 0.17 would be significant for the models. For each regression model, the predictor was level of gaming risk. Participant gender, age, and free/reduced-price lunch status were the control variables. Outcomes included two overarching categories: (a) behavioral health (i.e., depressive symptoms, hours of sleep, time spent on the computer, problematic internet behaviors, and (b) online social interactions (i.e., frequency of online game play, playing games alone, playing games with strangers, and frequency of checking social media). Regression analyses were used to investigate associations of gaming risk on behavioral health and online social interactions, which included transforming a categorical variable into an ordinal value into the linear regression [38]. Each participant was assigned a value associated with the game they have ever played with the highest gaming risk. For example, if a student reported playing Minecraft and Mortal Kombat, they were assigned a 3. Dummy variables for each level of gaming risk were used in the model with “no gaming risk” or “non-gamers” as the reference category. Adolescent gender, age, and free/reduced-price lunch (proxy for socioeconomic status) were used as control variables. Previous studies have found that individual-level and community-level factors such as gender [39], age [40], and socioeconomic status [41] are highly associated with how problematic gaming or digital media use influences wellbeing. All available data for each outcome were used in the regression models. For modeling frequency of online game play and who gamer plays with, only those who reported playing online games were included (i.e., non-gamers were automatically skipped out of answering these game-based questions through Qualtrics) and the “minimal risk” group was used as the comparison category.

## 3. Results

Seven hundred student surveys were collected. All surveys were used for analysis, but some surveys had missing data for the outcomes, resulting in a range of sample sizes from *n* = 381 to 609. With a response rate ranging from 88 to 92% from each site, participants were ethnically/racially diverse (52% female, 48% White, 16% Asian, 14% Black, 11% Latino, 11% Biracial/Other) early adolescents with a mean age of 12.7 (range = 11–16). A total of 32% were in grade 6, 30% in grade 7, and 38% in grade 8. A total of 77% reported that they received mostly As or As and Bs in the past year. Twenty-six percent were eligible for free or reduced-priced school lunch programs. In the following section, we will identify participants by their highest game risk level, such that middle school students who did not play any games will be called “non-gamers” (31%), students who only played games that had a minimal risk rating of “1” will be called “minimal-risk gamers” (17%). Middle school students who had ever played a game with a “2” rating will be called “moderate-risk gamers” (18%), and those who had ever played a level “3” game will be called “high-risk-gamers” (33%) (see Table 1). A total of 9% of players reported gaming for 3 h or more on a typical school night.

### Gaming Risk

Moderate-risk gamers reported higher problematic internet behaviors than participants who did not play games. Moderate-risk gamers were more likely to play online games with others than minimal-risk gamers. High-risk gamers reported higher depressive symptoms and problematic internet behaviors, less sleep and higher frequency of checking social media than non-gamers. High-risk gamers also spent more time playing games, were less likely to play alone, and more likely to play with unknown players than minimal-risk gamers (see Table 2).

## 4. Discussion

These results demonstrate that for early adolescents who play mainly minimally risky games, there was some risk of problematic internet behaviors but no other behavioral health and online social withdrawal. Moderate-risk and high-risk gamers were significantly more likely to play with strangers. Prior research suggests that playing with strangers increases the likelihood of having IGD [34]. It was only when compared to non-gamers that the high-risk gaming level was associated with more physical and mental health difficulties, such as less sleep and more depressive symptoms. Scholars have demonstrated that internet use is often used as a form of mood regulation, such as relieving feelings of boredom, loneliness, depression, or providing an escape option [42]. LaRose and colleagues [43] further suggest that using the internet to relieve feelings of depression can lead to deficient self-regulation, which may explain the sleep outcomes in the current study. Interestingly, high-risk gamers showed a greater likelihood of increased online social media interactions. Currently, there are many online games that offer ample opportunities for interactions with peers through text messaging or voice chat, which facilitates strategizing during a game, thereby fostering online friendships and moving toward mutually defined gaming goals [44]. As we demonstrated in the current study, the high-risk gamers were connected to their social media friends even beyond the gaming environment [25].

A limitation of this study was the identification of gaming risk level using the criteria of ever having tried the game, which does not account for frequency or recency of having played any particular game. Future research should measure gaming content risk level by most frequently and/or most recently played games. Future studies should replicate our findings with a more current sample, given the evolution of gaming culture. For instance, during the data collection of the current study, the widely popular game Fortnite debuted in fall 2017, a phenomenon that may have had a social impact in adolescent gaming culture.

## Study 2

## 5. Materials and Methods

### 5.1. Data Collection

Using a different school-based sample in the Northeast US than Study 1, we recruited 1 large suburban middle school and 3 urban afterschool programs to participate in a study of digital media use, including social media, YouTube, and gaming in the period 2018–2019. Similarly to Study 1, school and site selection was based on racial/ethnic composition, urbanicity, and socioeconomic diversity. The study was named Adolescent Social Media Use, Health, and Parental Monitoring. After obtaining IRB approvals from our institution and school districts to conduct the convenience sampling study of 6th–8th grade students using a waiver of consent documentation, we distributed parent informed consent/opt-out forms in English, Spanish, and Portuguese through parent email listservs and school enewsletters. The middle school provided Chromebooks during a pre-scheduled advisory period to take the 40 min survey. The afterschool programs provided Chromebooks during scheduled break times. Since survey links were emailed to students, those who were absent during survey administration were still able to participate in the study from home. Raffle prizes to a select few and embossed pens were given to every student during survey administration. Schools were also given an aggregated report of findings and an honorarium for participating in the study.

### 5.2. Measures

Sociodemographic variables included gender, age, and free/reduced-price lunch, similar to Study 1.

*Gaming risk level*. We updated the gaming risk categories to include the new games students reported playing that were not already identified in Study 1. Categories were created using the same industry standard metrics in Study 1 above. To maximize validity of responses, in Study 2, we asked respondents to report only the top three games that they play most frequently in order to determine their gaming risk level. This improves on the lack of specificity in Study 1 to include only the three most frequently played rather than a lengthy checklist of having ever having played a game.

#### 5.2.1. Behavioral Health

*Sleep duration*. Participants were asked on average how many hours of sleep they get on a typical school night. Responses ranged from 4 h or less to 10 or more hours. On average, participants reported getting approximately 7.5 h of sleep on a typical night.

*Bedtime*. Participants were asked by what time they fell asleep on a typical school night, with responses ranging from 9pm or earlier to 2am or later. Participants were, on average, getting to bed at approximately 10pm.

*Depressive symptoms*. Symptoms of depression were assessed using the Center for Epidemiologic Studies Depression Scale Revised (CESD-10), a 10-item Likert scale [45] that measures how often participants felt depressive symptoms in the past week. Response choices were “not at all or less than 1 day”, “1–2 days”, “3–4 days”, or “5–7 days”. A score of 10 or higher indicates the presence of significant depressive symptoms. A total of 19% of the sample would be considered depressed according to this cutoff score.

#### 5.2.2. Online Social Interactions

*Online gaming frequency*. Participants reported how often they play online games on a typical school day. Responses were reported on a 5-point scale ranging from “I don’t play online games” (lowest value) to “8 or more hours” (highest value). The average online games play time for the full sample is 2.38 (SD = 2.34), which is equivalent to approximately 2 h of play on a typical school day.

*Online gaming interactions*. Participants were asked a single-item measure of how often they interact with other players or chat while gaming. Response options were on a 5-point scale from never to always. A total of 46% reported they often or always interact with other players while gaming.

*Money spent on monthly gaming*. Participants were asked how much they spent on games that they played in a typical month, including on accessories and Vbucks. Amounts ranged from $0 to $100 or more. On average, 61% spent nothing in a typical month, 19% spent up to $10, 11% spent up to $25, 5% spent up to $50, 1% spent up to $100, and 3% spent over $100/month.

*Social withdrawal due to gaming*. On a 5-point scale ranging from 1 (never), 2 (once or twice), 3 (3–6 times), and 4 (more than 6 times), participants were instructed to indicate whether they had done the following in the past two weeks due to their gaming: (a) spent less time on homework or class projects (43%), (b) spent less time with family (42%), (c) skipped a meal (14%), (d) missed a sports or afterschool activity (6%), or (e) missed or was late to school (6%).

### 5.3. Data Analysis

Similar to Study 1, after data collection, we then recoded the gaming risk levels in a separate procedure conducted by undergraduate research assistants who were blinded to which study participants played which games. For each regression model, the predictor was level of gaming risk. Participant gender, age, and free/reduced-price lunch status were the control variables. Outcomes were under two categories: (a) *behavioral health* (i.e., depression symptoms, hours of sleep, and bedtime), and (b) *online social interactions* (i.e., frequency of online game play, interacting with other players while gaming, money spent while playing online games, social withdrawal due to gaming). Linear regressions were used to assess associations between gaming risk and behavioral health and social interactions outcomes. The “non-gamers” group was used as the reference category for non-gaming-related outcomes (i.e., hours of sleep and bedtime) and the “minimal risk” was the reference category for the gaming-related outcomes (e.g., who gamers play with). All models were conducted using SPSS and all data available were used to model outcomes. Sample sizes ranged from (N = 625–665) for the analysis models.

## 6. Results

**Figure 1:**
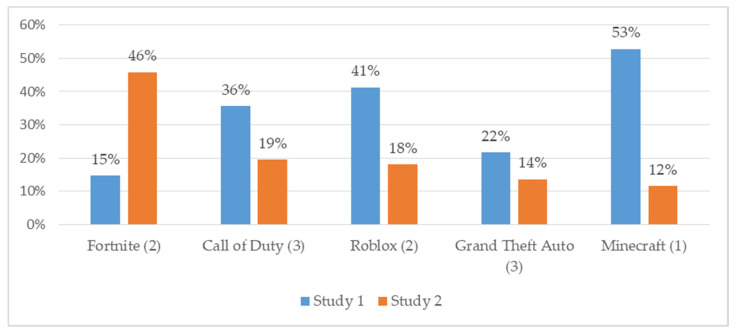
Top 5 most popular games played (risk level).

Surveys were collected from 799 participants. Fifteen student surveys were removed since they only gave assent, one respondent did not agree to take the survey, and 11 were duplicates. Participants in the analysis sample were 772 ethnically/racially diverse (50% female, 57% White, 18% Latino, 12% Black, 5% Asian, and 8% Biracial/other) early adolescents with a mean age of 12.6 (range = 11–15 years). A total of 33% of the sample were in grade 6, 35% in grade 7, and 32% in grade 8. A total of 24% reported being eligible for free or reduced-price school lunch programs. A total of 74% reported receiving mostly As or As and Bs in the past year. This sample had 5% non-gamers, 24% minimal-risk gamers, 33% moderate-risk gamers, and 38% high-risk gamers. A total of 35% of the sample reported playing games on average three or more hours on a typical school night. A total of 9% of gamers spent $50–100+ per month on in-game purchases (see Table 3). Compared to the Study 1 sample, Study 2 comprised of more gamers overall and more high-risk gamers. Figure 1 illustrates the top 5 games played by participants in Study 1 and Study 2.

### Gaming Risk

There was no significant association found between depression and gaming risk level. High-risk gamers reported fewer hours of sleep per night and a later bedtime than non-gamers (see Table 4). Moderate- and high-risk gamers report spending more time playing online games, interacting more while playing, and spending more money while gaming than minimal-risk gamers. Moderate- and high-risk gamers more frequently reported spending less time on homework and less time with family than minimal-risk gamers. High-risk gamers also more frequently reported skipping meals. There was no association between risky gaming level and having missed sports or afterschool activity.

## 7. Discussion

Moving beyond associations between health, wellbeing, and frequency of gaming behaviors, Study 2 explored the everyday social consequences of online gaming, that is, the extent to which the content of gaming interfered with daily adolescent life, particularly social withdrawal. Compared to the lowest-risk gamers, the moderate- and high-risk gamers experienced similar levels of online social interactions, gaming frequency, and gaming spending habits. More research is needed to understand parents’ role in allowing in-game purchasing or whether vulnerable users are being financially and psychologically exploited by the gaming industry. Although our study found that 39% of early adolescent gamers spend some money on gaming accessories and Vbucks, with 4% spending approximately $100 and up per month, little is known about whether early adolescents are aware of how online microtransaction tactics that games use to pressure them into “limited time” purchases [46]. More research on gaming spending habits may help families flag the types of gaming content that are associated with higher spending tendencies, which may be a potential concern for families.

Moderate- and high-risk gamers had more educational and family-related withdrawal, whereas the highest-risk gamers also experienced consequences related to self-care (sleep duration, bedtime, and skipping meals), signaling a tendency to more problematic gaming behaviors. Interestingly, risky gaming content was not associated with withdrawing from one’s extracurricular activities, but rather affected educational and family interactions mainly. This may be due to the desire to maintain social connections with peers both online and offline. Given prior research indication that improving family cohesion during therapy helped to treat problematic gaming [47], paying attention to the socializing environment [48], such as family mealtimes and other opportunities to spend time together could help mitigate potentially problematic gaming habits. Playing high-risk games was associated with foregoing family and self-care activities, which raises the question of whether the online gaming is simply preferred over those other activities or whether the social interactions from gaming fulfill their social needs in ways that in-person activities do not. A preference for online social interactions (POSI) may be one reason why frequent gamers are often the most socially interactive compared to infrequent gamers [49]. Another explanation could be the heightened sense of online social support that is received during collaborative gaming context, particularly in violent gaming contexts. Prior research on the effects of violent gaming on physiological arousal has demonstrated that active online interactions during violent gaming tasks significantly reduces the mental and physical load on players compared to those playing solo [50].

## 8. Conclusions

We explored the social and behavioral risk factors of age-inappropriate gaming in two samples in the Northeast US using slightly different methodologies that captured the rapidly evolving gaming ecologies of that time. We redefined risk level of game playing according to frequency of use (rather than ever having played a risky game in Study 1), conducting a similar analysis while adding additional constructs related to social withdrawal impacts of problematic gaming. Study 1 was a sample conducted in the period 2017–2018 around wherein one-third of the sample were non-gamers, only 9% reported gaming over 3 h/week night, and the massively popular game Fortnite (coded as a Medium-Risk game in our studies) had just debuted in the fall of that year (15% reported playing Fortnite). The sample for Study 2 had similar demographic characteristics conducted in different school settings, but only 5% reported being non-gamers, over one-third (35%) reported gaming over 3 h/week night, and by this time in the period 2018–2019, almost half of the gaming sample (46%) reported Fortnite as one of the top three most frequent games that they played (a three-fold increase since Study 1). Despite the differences in the sample characteristics, we demonstrated that many early adolescents who are playing online games that are meant for mature audiences, such as medium risk (e.g., Fortnite) and high-risk (e.g., Call of Duty) games, are more likely to experience symptoms of addictive behaviors and have problems with mental wellbeing. For instance, those playing the riskiest games tend to get fewer hours of sleep, a deprivation that can result in problems with attention, memory, decision making, reaction time, and creativity, all of which are important to achieving in school. A higher amount of screen-based activities, including gaming, has been associated with more problems falling asleep and shorter weekday duration [51]. Prior research has also demonstrated that the existence of computers, TVs, and gaming consoles in a child’s bedroom doubles the amount of time they play each week and the likelihood of playing mature games [51]. This finding suggests that parents can make a difference in the potential negative impacts of gaming by restricting the timing and location of gaming at bedtime.

Not only are parents a primary socializer for adolescents, they are usually the first ones to be able to notice a persistent change in behavior or mood due to gaming, and possibly identify in what ways gaming has affected their child’s social and family life. For instance, parents might first suspect a problem when adolescents aren’t sleeping regularly with the introduction of a new game, are having trouble completing their homework or school projects, are losing their appetite and missing meals. This first-hand observation is critical when parents decide to turn to pediatricians, educators, and counselors for advice on whether the problem is a passing phase or whether it is something to intervene before it progresses too far.

Due to the cross-section designs of both studies, we cannot determine the direction of influence in the associations between gaming, behavioral health, and online social interactions. Although our analysis plans were not pre-registered, as is recommended to prevent false positive findings [52], we have provided detailed procedures for both of the exploratory studies presented in this article. One of the strengths of reporting on multiple studies is the opportunity to present analyses that build off of one another. For instance, Study 1 focused on social interactions and Study 2 focused on social withdrawal behaviors due to gaming. Additionally, we presented all findings within the behavioral health (e.g., sleep and depression) and gaming-related social interactions or withdrawal constructs in our dataset and used the exact same covariates for both studies. Another limitation to note is the self-reporting nature of the sleep and behavioral health outcomes that may be subject to mono-informant bias [51]. Due to the listwise deletion of all cases with missing data on variables of interest, estimates may be less efficient due to resulting loss of information [53]. However, our response rates of over 80% support external validity of our data. Potential confounders in interpreting the relationship between gaming risk and behavioral health include the media context within the household, (e.g., presence of smartphones in the bedroom), parental monitoring style, and family structure, (e.g., having an older sibling who may introduce more mature games to siblings at younger ages). Future research can build on these findings to advance our understanding of any causal relationships as well as which subgroups are more vulnerable when playing the higher-risk games. Since mature or age-inappropriate games can be associated with relatively mild problems to deleterious outcomes that are considered more severe, using a continuum approach rather than a categorical approach may be more practically useful to evaluate how and when to intervene to prevent exacerbating the dilemma [54]. As research continues to examine both adaptive and maladaptive gaming through measuring levels of distress or clinical diagnoses, it is also important to discuss the age appropriateness and maturity level of the gaming content consumed when advising patients and students in clinical, educational, and therapeutic practices.

## Figures and Tables

**Table 1:** Study 1: Sample Descriptives.

Variable		N	Percent
Gender			
	Female	364	52.0
	Male	331	47.3
	Other	5	0.7
Race			
	White	328	47.7
	Asian	110	16.0
	Black	97	14.1
	Latino	77	11.2
	Biracial/Other	75	10.9
Grade			
	6th	223	31.9
	7th	214	30.6
	8th	263	37.6
Grades in school			
	Mostly As	288	42.3
	Mostly As and Bs	237	34.8
	Mostly Bs	51	7.5
	Mostly Bs and Cs	73	10.7
	Mostly Cs	22	3.2
	Mostly Ds or Fs	10	1.5
Free/reduced-price lunch		109	25.8
Gaming risk			
	Non-gamers	212	31.3
	Minimal risk	118	17.4
	Moderate risk	122	18
	High risk	226	33.3
Frequency of game play			
	Less than 30 min/day	199	43.0
	30–60 min	125	27.0
	1–2 h	78	16.8
	3 h+	61	13.2
Plays games alone		250	53.6
Plays games with strangers		157	33.7
Age		699	12.70 (1.00)
Depressive symptoms		475	1.69 (0.61)
Hours of sleep		457	8.06 (1.22)
Computer time		428	1.78 (0.74)
Problematic internet use		688	1.78 (0.70)
Frequency of checking social media		672	3.20 (2.41)

**Table 2:** Study 1: Gaming Risk on Adolescent Behavioral Health and Online Social Interactions.

Predictor	Behavioral Health							
	Depressive Symptoms	Hours of Sleep	Computer Time	Problematic Internet Use	Freq of Game Play	Plays Games Alone	Plays Games With Strangers	Frequency of Checking Social Media
	B (SE)	B (SE)	B (SE)	B (SE)	B (SE)	B (SE)	B (SE)	B (SE)
Min Risk	0.10 (0.08)	0.10 (0.17)	0.05 (0.11)	0.14 (0.08)	--	--	--	−0.19 (0.27)
Mod Risk	0.02 (0.09)	−0.05 (0.17)	0.16 (0.11)	0.18 (0.08) *	0.20 (0.14)	−0.61 (0.30) *	0.65 (0.35)	0.07 (0.27)
High Risk	0.18 (0.08) *	−0.34 (0.16) *	0.13 (0.10)	0.30 (0.08) ***	0.42 (0.13) **	−1.14 (0.29) ***	0.75 (0.33) *	0.99 (0.24) ***
Female	0.14 (0.06) *	−0.19 (0.13)	−0.09 (0.08)	0.13 (0.06) *	−0.51 (0.11) ***	0.26 (0.22)	−1.23 (0.25) ***	1.01 (0.20) ***
Age	0.05 (0.03)	−0.25 (0.06) ***	0.12 (0.04) **	0.04 (0.03)	0.04 (0.05)	−0.08 (0.10)	0.16 (0.11)	0.79 (0.09) ***
Free lunch	0.04 (0.09)	−0.45 (0.19) *	0.07 (0.12)	0.06 (0.08)	0.50 (0.13) ***	−0.56 (0.28) *	0.03 (0.29)	0.66 (0.24) **
R^2^	0.03	0.10	0.05	0.03	0.16	0.11	0.16	0.20

Note: Non-gamers was the comparison category used for non-gaming-related outcomes. Minimal risk was the comparison category for gaming-related outcomes. * *p* < 0.05, ** *p* < 0.01, and *** *p* < 0.001.

**Table 3:** Study 2: Sample Descriptives.

Variable		N	Percent
Gender			
	Female	384	49.7
	Male	379	49.1
	Other	9	1.2
Race			
	White	414	56.6
	Asian	38	5.2
	Black	86	11.8
	Latino	134	18.3
	Biracial/Other	59	8.0
Grade			
	6th	250	32.5
	7th	273	35.3
	8th	248	32.2
Grades in school			
	Mostly As	272	35.5
	Mostly As and Bs	293	38.3
	Mostly Bs	67	8.7
	Mostly Bs and Cs	91	11.9
	Mostly Cs	25	3.3
	Mostly Ds or Fs	18	2.3
Free/reduced-price lunch		178	23.5
Frequency of game play			
	Less than 60 min	215	36.1
	2 h	127	21.3
	3 h	92	15.5
	4+ hours	161	27.1
Money spent			
	$0	422	61.1
	Up to $10	127	18.4
	Up to $25	79	11.4
	Up to $50	35	5.1
	Up to $100	8	1.2
	$100 or more	20	2.9
Bedtime			
	9pm or earlier	196	29.4
	10pm	267	40.0
	11pm	140	21.0
	12am	40	6.0
	1am or later	24	3.6
Gaming risk			
	Non-gamers	40	5.4
	Minimal risk	176	23.9
	Moderate risk	240	32.7
	High risk	279	38.0
Age		772	12.57 (0.96)
Depressive symptoms		649	6.28 (4.98)
Hours of sleep		673	7.51 (1.38)
Interacts with other players		693	3.06 (1.46)
Spend less time on HW		687	1.56 (0.76)
Spend less time with family		681	1.60 (0.85)
Skipped a meal		677	1.21 (0.60)
Missed sports or afterschool activity		678	1.09 (0.41)

**Table 4:** Study 2: Gaming Risk on Adolescent Health and Online Social Interactions.

	Behavioral Health				Online Social Interactions					
	Depression Symptoms	Hours of Sleep	Bedtime	Freq of Game Play	Interact with Other Players	Money Spent	Spend Less Time on HW	Spend Less Time with Family	Skipped A Meal	Missed Sports or After-school Activity
	B (SE)	B (SE)	B (SE)	B (SE)	B (SE)	B (SE)	B (SE)	B (SE)	B (SE)	B (SE)
Min Risk	−1.25 (0.89)	−0.03 (0.25)	0.15 (0.18)	--	--	--	--	--	--	--
Mod Risk	0.79 (0.89)	−0.08 (0.25)	0.28 (0.19)	1.06 (0.24) ***	1.05 (0.13) ***	0.37 (0.12) **	0.23 (0.08) **	0.19 (0.09) *	0.11 (0.06)	0.04 (0.04)
High Risk	1.04 (0.92)	−0.61 (0.26) *	0.51 (0.19) **	1.87 (0.27) ***	1.43 (0.15) ***	0.88 (0.13) ***	0.36 (0.09) ***	0.42 (0.10) ***	0.22 (0.07) **	0.08 (0.05)
Female	3.17 (0.48) ***	−0.66 (0.13) ***	0.37 (0.10) ***	−0.34 (0.21)	−0.75 (0.11) ***	−0.40 (0.10) ***	0.00 (0.07)	−0.01 (0.08)	0.06 (0.06)	−0.01 (0.04)
Age	−0.26 (0.20)	−0.30 (0.06) ***	0.24 (0.04) ***	−0.11 (0.09)	−0.07 (0.05)	−0.07 (0.04)	−0.03 (0.03)	−0.05 (0.03)	−0.03 (0.02)	−0.02 (0.02)
Free lunch	1.42 (0.46) **	−0.22 (0.13)	0.12 (0.09)	0.00 (0.21)	−0.22 (0.11) *	−0.07 (0.10)	0.09 (0.07)	−0.03 (0.08)	0.06 (0.06)	0.10 (0.04) **
R^2^	0.09	0.10	0.08	0.14	0.33	0.18	0.04	0.04	0.02	0.02

Note: Non-gamers was the comparison category used for non-gaming-related outcomes. Minimal risk gaming was the comparison category for gaming-related outcomes. * *p* < 0.05, ** *p* < 0.01, and *** *p* < 0.001.

## Appendix A

**Table A1:** Games Played by Participants in Study 1 and 2, Percent Played, and Risk Levels.

Game	% Study 1	% Study 2	Minimum Age (Common Sense)	Violence (/5)	Substance Use (/5)	Sex (/5)	Language (/5)	ESRB Rating (E, E 10+, T, M, A)	Risk Level (/3)
Angry Birds	37.3	0.5	7+	2	0	0	0	E	1
Assassin’s Creed	--	2.0	18+	4	0	0	4	M	3
Battlefield	--	2.0	18+	5	0	0	3	M	3
Call of Duty	35.6	19.5	18+	5	0	0	3	T/M	3
Candy Crush Saga	--	4.5	13+	0	0	0	0	--	1
Fallout	14.0	1.4	16–18+	3 to 5	3 to 4	4	4	M	3
Flow Free	13.6	2.2	8+	0	0	0	0	--	1
Fortnite	14.6	45.9	13+	3	0	0	1	T	2
Forza	--	1.6	10–13+	2	0	0 - 2	3	E / E 10+	2
Geometry Dash	--	3.8	8+	1	0	0	0	--	1
Grand Theft Auto	21.6	13.5	18+	5	5	5	5	M	3
Happy Glass	--	3.0	8+	0	0	0	0	12+	1
Helix Jump	--	13.4	9+	0	0	0	0		1
Homescapes	--	1.5	7+	0	0	0	0	4+	1
Just Dance	34.7	7.3	11+	0	3	3	1	E / E 10+	1
Lego Dimensions	4.7	0.5	10+	2	0	0	1	E 10+	1
Lovers in a Dangerous Spacetime	--	0.1	12+	1	0	0	0		2
Medal of Honor	3.0	0.1	18+	4	0	0	5	M	3
Minecraft	52.8	11.5	8–10+	2	0	0	0	E 10+	1
Mortal Kombat	19.3	3.1	18+	5	0	3	4	M	3
NHL/NBA/Madden NFL/Wii Sports	--	14.2	8+ to 11+	1–3	0	0	0	E	1
Overwatch	--	4.9	13+	3	1	2	0	T	2
Pokémon	--	3.0	7+	2	0	1	0	E / E 10+	2
Portal	10.2	0.4	12+	3	0	0	0	T	2
Rainbow Six: Siege	--	7.7	18+	4	0	0	4	M	3
Red Dead Redemption	--	2.3	18+	5	5	5	5	M	3
Roblox	41.3	18.0	10+	4	0	0	1	E 10+	2
Rolling Sky	32.6	7.0	8+	0	0	0	0	--	1
Subway Surfers	--	12.2	9+	2	0	0	0	--	1
Super Smash Brothers Ultimate	--	4.5	7–12+	0 to 2	0	0 to 1	0	E / E 10+	1
Temple Run	48.7	4.9	9+	3	0	0	0	--	1
Terraria	8.5	0.4	11+	3	1	0	1	T	2
Zoo Tycoon	3.0	0.0	10+	0	0	0	0	E	1

Note: We began with 16 most popular games that teens played for Study 1. We added the missing games that were popular in Study 1 as “write-in” games in Study 2, which increased the list to 33.

## Appendix B. Measures for Study 1 and Study 2

  *Gender*

Are you…? Male Female  Other (male transgender, female transgender, non-binary)

  *Age*

What is your birth month and day?

What year were you born?

* Free or reduced-price lunch*

Do you get free or reduced price lunch at your school?

Yes No Don’t know

  *Depression symptoms* (see Houghton et al., 2006; Haroz, Ybarra, & Eaton, 2014 for Study 1; see Björgvinsson et al., 2013 for Study 2)

  *Sleep Duration*

On an average school night, how many hours of sleep do you get?

○ 4 or less hours	○ 8 h
○ 5 h	○ 9 h
○ 6 h	○ 10 or more hours
○ 7 h	

  *Bedtime (Study 2 only)*

By what time do you usually fall asleep on a typical school night?

○ 9pm or earlier
○ 10pm
○ 11pm
○ 12am
○ 1am or later

  *Recreational Computer Use (Study 1 only)*

On an average school day, how many hours do you use a computer for something that is not school work? (Count time spent on Xbox, PlayStation, iPod, tablet, smartphone, YouTube, Facebook, browsing the Internet.)

○ I do not use a computer for something that is not school work
○ Less than 1 h per day
○ 1 h per day
○ 2 h per day
○ 3 h per day
○ 4 h per day
○ 5 or more hours per day

  *Problematic Internet Behaviors* (see Moreno, Arseniev-Koehler, & Selkie, 2016 for Study 1)

  *Games played (Study 1 only)*

Which online games have you played? (check as many as you want)

I don’t play online games	Portal
Minecraft	Fallout
Angry Birds	Temple Run
Mortal Kombat	Rolling Sky
Lego Dimensions	Flow Free
Medal of Honor	Roblox
Zoo Tycoon	Grand Theft Auto
Call of Duty	Terraria
Just Dance	List others

  *Games played (Study 2 only)*

In the past year, which of these games have you played the most? (select up to 3)

I don’t play games Flow Free   Happy Glass

Fortnite   Roblox    Homescapes

Minecraft  Grand Theft Auto  Assassin’s Creed 

Angry Birds  Mortal Kombat   Helix Jump

Medal of Honor  Lego Dimensions  Paper.io

Zoo Tycoon  Terraria    NHL/NBA/Madden/NFL/wii sports

Call of Duty  Super Smash Brothers Ultimate Battlefield

Just Dance  Overwatch   Geometry Dash

Portal   Pokemon   Red Dead Redemption

Fallout   Subway Surfers   Forza

Temple Run  Rainbow Seige   Lovers in a Dangerous Spacetime

Rolling Sky  Candy Crush   List others_____________________

  *Online gaming frequency (Study 1 only)*

How often do you play online games on a typical school day?

○ I don’t play online games
○ Less than 10 min/day 10-30 min
○ 30–60 min
○ 1–2 h
○ 3 h +

  *Online gaming frequency (Study 2 only)*

On an average school day, up to how many hours do you play games (include online, phone, Xbox, Playstation, etc.)

○ Never
○ Up to 1 h
○ 2 h
○ 3 h
○ 4 h
○ 5 h
○ 6 h
○ 7 h
○ 8 h
○ More than 8 h

  *Online gaming interactions (skipped if checked “I don’t play online games” above, Study 1)*

Who do you usually play online games with? (check any that apply)

○ I usually play alone or against no one	○ Adults I know, like parents or relatives
○ Kids I know, like friends or siblings	○ Adults I have never met before
○ Kids I have never met before	○ I have no idea who I play with online.

  *Online gaming interactions (skipped if checked “I don’t play online games” above, Study 2)*

How often do you interact with other players or chat while you are gaming?

○ Never
○ Rarely
○ Sometimes
○ Often
○ Always

  *Social media frequency (Study 1 only)*

On a typical school week, how often do you check your social media (like Instagram)

○ More than every hour   A few times a week
○ Every few hours   Less often than a few times a week
○ Once a day    Never/Does not apply to me
○ Every few days

*Money spent on monthly gaming (Study 2 only)*

In a typical month, how much do you spend on games you play (includes accessories, Vbucks, etc.)?

○ $0
○ Up to $10
○ Up to $25
○ Up to $50
○ Up to $100
○ $100 or more

*Social withdrawal due to gaming (Study 2 only)*

In the past 2 weeks, how often have you done the following because of the games you’ve played?

	Never	Once or twice	3–6 times	More than 6 times
Spent less time on homework or class projects	○	○	○	○
Spent less time with family	○	○	○	○
Skipped a meal	○	○	○	○
Missed a sports or afterschool activity	○	○	○	○
Missed school/late to school	○	○	○	○

Note: Once the datasets are no longer active, we will provide the data upon request.
